# Association between low total serum testosterone and body mass index in Australian survivors of testicular cancer: a retrospective analysis

**DOI:** 10.1186/s12610-024-00230-5

**Published:** 2024-09-03

**Authors:** Grace Y. Kim, Ciara Conduit, Sophie O’Haire, Chia Yuen Chong, Olivia Baenziger, Jeremy Lewin, Benjamin Thomas, Nathan Lawrentschuk, Martin R. Stockler, Ian Olver, Peter Grimison, Ben Tran

**Affiliations:** 1 Walter and Eliza Hall Institute of Medical Research, Melbourne, VIC Australia; 2 Department of Medical Oncology, Peter MacCallum Cancer Centre, Melbourne, VIC Australia; 3 Sir Peter MacCallum Department of Oncology, The University of Melbourne, Parkville, VIC Australia; 4 ONTrac at Peter Mac Victorian Adolescent & Young Adult Cancer Service, Parkville, VIC Australia; 5 Department of Surgery, Royal Melbourne Hospital, University of Melbourne, Parkville, VIC Australia; 6 Department of Surgical Oncology, Peter MacCallum Cancer Centre, Melbourne, VIC Australia; 7 Epworth Freemasons Hospital, Melbourne, VIC Australia; 8 Australian and New Zealand Urogenital and Prostate (ANZUP) Cancer Trials Group, Barangaroo, NSW Australia; 9 National Health and Medical Research Council Clinical Trials Centre, University of Sydney, Camperdown, NSW Australia; 10 Concord Repatriation General Hospital, Sydney, NSW Australia; 11 Chris O’Brien Lifehouse, Sydney, NSW Australia

**Keywords:** Hypogonadism, Obesity, Testicular cancer survivors, Testosterone, Body mass index, Hypogonadisme, Obésité, Survivants du cancer des testicules, Testostérone, Indice de masse corporelle

## Abstract

**Background:**

Primary hypogonadism is a recognised complication in survivors of testicular cancer. However, secondary hypogonadism can result from other causes that suppress the hypothalamic-pituitary axis, including obesity, high dose glucocorticoids, chronic end organ failure, and diabetes. The aim of this study was to explore low total serum testosterone in Australian survivors of testicular cancer and examine associations with body mass index, age, and prior chemotherapy use.

**Methods:**

Clinical data including height, weight, diagnosis, treatment, and hormonal evaluations during follow-up were extracted from the Australian and New Zealand Urogenital and Prostate (ANZUP) Cancer Trials Group Chemocog study (2007-2012), accompanied by data from two Australian, high-volume testicular cancer centres included in the iTestis testicular cancer registry (2012-2019). Low testosterone was defined by a serum concentration of testosterone (T) < 10 nmol/L, and was classified as primary by a serum concentration of luteinising hormone (LH) > 8 IU/L, otherwise as secondary.

**Results:**

Two hundred eighty-five individuals with either stage 1 or advanced testicular cancer were included. Of these, 105 (37%) were treated with orchidectomy and chemotherapy. Forty-nine (17%) met criteria for low testosterone during follow-up: 21 (43%) had primary and 27 (55%) had secondary low testosterone. Survivors of testicular cancer with higher body mass index were more likely to display low testosterone, both primary (*p* = 0.032) and secondary (*p* = 0.028). Our data did not show evidence of an association between older age or chemotherapy use and low testosterone in our cohort.

**Conclusions:**

Low total serum testosterone was common in survivors of testicular cancer, and associated with a higher body mass index prior to orchidectomy, suggesting that elevated body mass index may contribute to low testosterone in this population, and that body weight, diet, and exercise should be addressed in testicular cancer follow-up.

## Résumé

**Contexte:**

L’hypogonadisme primaire est une complication reconnue chez les survivants d’un cancer du testicule. Cependant, l’hypogonadisme secondaire peut résulter d’autres causes qui suppriment l’axe hypothalamo-hypophysaire, notamment l’obésité, les glucocorticoïdes à forte dose, la défaillance chronique des organes cibles et le diabète. Le but de cette étude était d’explorer un faible taux de testostérone totale sérique chez les survivants australiens d’un cancer du testicule, et d’examiner les associations avec l’indice de masse corporelle, l’âge et l’utilisation antérieure d’une chimiothérapie.

Les données cliniques, y compris la taille, le poids, le diagnostic, le traitement et les évaluations hormonales au cours du suivi, ont été extraites de l’étude Chemocog de l’Australian and New Zealand Urogenital and Prostate (ANZUP) Cancer Trials Group (2007–2012), accompagnées de données, provenant de deux centres australiens à fort volume de prise en charge de cancers du testicule, incluses dans le registre du cancer du testicule iTestis (2012–2019). Un taux faible de testostérone a été défini par une concentration sérique de testostérone (T) < 10 nmol/L, et a été classé comme primaire pour une concentration sérique d’hormone lutéinisante (LH) > 8 UI/L, sinon comme secondaire.

**Résultats:**

Deux cent quatre-vingt-cinq personnes atteintes d’un cancer des testicules de stade 1 ou avancé ont été incluses. Parmi ceux-ci, 105 (37%) ont été traités par orchidectomie et chimiothérapie. Quarante-neuf (17%) répondaient aux critères d’un taux faible de testostérone au cours du suivi: 21 (43%) avaient un taux faible de testostérone primaire et 27 (55%) un faible taux secondaire. Les survivants d’un cancer du testicule avec un indice de masse corporelle plus élevé étaient plus susceptibles de présenter un taux faible de testostérone, à la fois primaire (*p* = 0,032) et secondaire (*p* = 0,028). Nos données n’ont pas montré de preuve d’une association entre un âge avancé ou l’utilisation de la chimiothérapie, et un taux faible de testostérone, dans notre cohorte.

**Conclusions:**

Un faible taux de testostérone sérique totale était fréquent chez les survivants d’un cancer du testicule, et associé à un indice de masse corporelle plus élevé avant l’orchidectomie; ceci suggère qu’un indice de masse corporelle élevé peut contribuer à un faible taux de testostérone dans cette population, et que le poids corporel, l’alimentation et l’exercice devraient être pris en compte dans le suivi du cancer du testicule.

## Background

Testicular cancer is the most common solid organ malignancy diagnosed in males aged 15–35 years [1]. Treatment of testicular cancer, depending on stage and risk stratification, includes surgery, chemotherapy, radiotherapy, or combinations of these [2]. Cisplatin-based chemotherapy has rendered testicular cancer highly curable with 10-year survival rates above 95% [1]. As such, most patients become long-term survivors, and addressing late treatment-related complications is an important aspect of their care.

Several studies have indicated that survivors of testicular cancer are at an increased risk of long-term treatment-related sequelae, including peripheral neuropathy, nephrotoxicity, ototoxicity, pulmonary toxicity, infertility, hypogonadism, osteoporosis, metabolic syndrome, anxiety/depression, and chronic fatigue [3–11]. Additionally, some survivors of testicular cancer may face potentially life-threatening late effects during long-term follow-up, including the development of secondary malignancies and cardiovascular disease [8–12]. In this study, we focus on hypogonadism as an important long-term complication of testicular cancer.

Hypogonadism is associated with reduced sexual function, low mood, osteoporosis, and increased risk of cardiovascular disease. Hypogonadism has been reported to occur in up to 40% of survivors of testicular cancer, which is substantially more common than in age-matched controls from the general population [13, 14]. Risk factors for the development of hypogonadism after treatment of testicular cancer include orchidectomy (both unilateral and bilateral), chemotherapy and infra-diaphragmatic radiotherapy [15, 16]. Individuals receiving multimodal or multiple lines of therapy are more likely to develop primary hypogonadism [17]. Another hypothesised mechanism for primary hypogonadism among survivors of testicular cancer is the testicular dysgenesis syndrome, where the tumour itself may cause hypogonadism from Leydig cell dysfunction [16].

The extent to which secondary hypogonadism may contribute to the prevalence of hypogonadism in survivors of testicular cancer is unknown. Secondary hypogonadism results from suppression of the hypothalamic-pituitary axis, which may be caused by obesity, high-dose glucocorticoids, chronic end organ failure, and diabetes. The extent to which the prevalence of these conditions among survivors of testicular cancer may contribute to the overall prevalence of hypogonadism has not been explored.

## Methods

The aim of this study was to describe the prevalence of low serum total testosterone in Australian survivors of testicular cancer and examine for possible associations with elevated body mass index (BMI), age, and prior chemotherapy use.

Clinical data were extracted from the Australian and New Zealand Urogenital and Prostate (ANZUP) Cancer Trials Group ‘Chemocog’ study [18, 19], which enrolled individuals aged 18 years or above, undergoing testicular cancer treatment from 16 centres across Australia and New Zealand between 2007–2012. This cohort included individuals with testicular cancer of any stage, including those on active surveillance, as well as those who had chemotherapy or radiotherapy.

To supplement the Chemocog sample, this dataset was amalgamated with data extracted from two centres within Australia’s national, multi-site, prospectively maintained testicular cancer registry, iTestis. iTestis, launched in 2017 and incorporating retrospective and prospective data, includes consecutive individuals with clinical stage 1 and advanced testicular cancer receiving follow-up care. At time of interrogation for this study, iTestis contained ~ 1000 patients diagnosed with clinical stage 1 and advanced testicular cancer from 15 Australian sites. Individuals diagnosed between 2012–2019 and aged 18 years or above as per the Chemocog cohort were included.

The final combined cohort comprised individuals aged 18 years or above, with any treatment history, who had at least two out of three available data points: pre-treatment BMI, post-orchidectomy testosterone, or LH.

Age at diagnosis, treatments, pre-orchidectomy or chemotherapy weight and height (taken at a single time point), and serum concentrations of testosterone and luteinising hormone (LH) taken between 4 to 18 months after orchidectomy were collected from Chemocog trial data and/or iTestis. Patients from iTestis had a single hormone measurement around 6 months post-orchidectomy as per ANZUP surveillance guidelines [20]. However, due to the real-world nature of the iTestis dataset, there were inconsistencies in the timing of the hormone measurements. Therefore, we accepted any measurements taken within 4–18 months post-orchidectomy as considered clinically relevant. In the Chemocog cohort, the majority of patients had multiple testosterone measurements (ranging from 2 to 6) within 4–18 months post-orchidectomy. To be consistent with the iTestis cohort, we selected the value closest to 6 months post-orchidectomy when multiple measurements were available. Data on other factors that contribute to secondary hypogonadism, such as steroid use, type 2 diabetes, and lifestyle factors, were not routinely collected in the iTestis registry and therefore not included in the analysis.

The data were summarised by the median for continuous variables and proportions for categorical variables. In addition to descriptive analysis, the Pearson’s chi-squared test (for categorical variables) and Wilcoxon rank sum test (for continuous variables) were used to examine associations between low total serum testosterone and age, prior chemotherapy use, and elevated BMI. The Cochran – Armitage test was also used to explore the association of low testosterone with increasing categories of BMI. BMI was calculated by dividing weight in kg by the squared height in meters. Low total serum testosterone was defined by a serum testosterone < 10 nmol/L as per the Endocrine Society of Australia guidelines [21], with LH > 8 IU/L distinguishing primary from secondary, based on National Health Service reference ranges for men > 14 years [22].

All statistical analyses were conducted using Stata version 14.0. All p-values and confidence intervals are 2-sided without adjustment for multiple comparisons. Analyses were conducted using pairwise deletion.

## Results

A total of 285 Australian survivors of testicular cancer were included in the final cohort, with 151 (53%) from Chemocog and 134 (47%) from iTestis. The median age at diagnosis was 33 years (range 18–64), and the median BMI was 26 kg/m^2^ (range 16–45 kg/m^2^); 53 individuals (23% of the cohort) had a BMI ≥ 30 kg/m^2^. Just over one-third of the cohort (37%) were treated with chemotherapy (Table 1).

**Table 1:** Summary of patient characteristics (*n* = 285)

	**Median (IQR)**^**a**^
**Age (years)**	33 (28–40)
**Body Mass Index (kg/m**^**2**^**)**	26 (24–29)
	**Patient number (%)**
**Treatment(s) received**	
Orchidectomy alone	180 (63%)
* Unilateral orchidectomy*	177 (98%)
* Bilateral orchidectomy*	5 (2%)
Orchidectomy + chemotherapy	105 (37%)
**Total serum testosterone < 10**	
Yes	49 (17%)
No	208 (73%)
Unknown (and excluded in subsequent analysis)	28 (10%)
**Types of low serum testosterone**	
Primary low testosterone (LH ≥ 8)^b^	21 (43%)
Secondary low testosterone (LH < 8)^b^	27 (55%)
Unknown (LH not available)^b^	1 (2%)

^a^*IQR* Interquartile range

^b^*LH* Luteinising hormone

Among the 285 eligible individuals, 28 (10%) had unknown testosterone levels and were excluded from further analyses. In the remaining 257 individuals, low total serum testosterone was diagnosed in 49 (19%), with primary low testosterone in 21 (43%) and secondary low testosterone in 27 (55%). One patient with low testosterone had unknown LH and could not be assigned to either group (Table 1).

A significant trend where individuals with higher BMI were more likely to have low testosterone was identified (*p* = 0.002) (Table 2), and this association was evident when assessing both primary (*p* = 0.032) and secondary (*p* = 0.028) low total serum testosterone (Table 3). While there was a higher proportion of low testosterone in patients treated with chemotherapy overall and in both the primary and secondary subgroups, these differences were not statistically significant. In our cohort, age was not associated with either primary or secondary low total serum testosterone.

Table 1 describes the baseline characteristics of the patients included in the analysis, including age, body mass index, treatment(s) received, and hormone measurements.

**Table 2:** Association of clinical factors with low serum testosterone (*n* = 257)

		**Normal testosterone (*n* = 208)**	**Low testosterone (*n* = 49)**	
**Clinical factors**				***P*-value**
Age in years, median (IQR)^d^		34 (29–40)	32 (28–39)	0.66^a^
Body Mass Index (kg/m^2^) (n, %)	< 20 (9, 4%)	9 (100%)	0 (0%)	0.002^b^
	20–24 (77, 32%)	71 (92%)	6 (8%)	
	25–29 (85, 33%)	63 (74%)	22 (26%)	
	≥ 30 (51, 19%)	38 (75%)	13 (25%)	
Treatment(s) Received (n, %)	Orchidectomy + Chemotherapy (90, 35%)	68 (76%)	22 (24%)	0.107^c^
	Orchidectomy alone (167, 65%)	140 (84%)	27 (16%)	

^a^Significance level determined by Wilcoxon rank-sum test

^b^Significance level determined by Cochran-Armitage test for trend

^c^Significance level determined by Pearson’s chi-squared test

^d^IQR- interquartile range

**Table 3:** Association of clinical factors with low serum testosterone according to primary low vs normal testosterone and secondary low vs normal testosterone

		**Normal testosterone (*n* = 208)**	**Primary low testosterone (*n* = 21)**		**Secondary low testosterone (*n* = 27)**	
**Clinical factor**				***P*-value**		***P*-value**
Age in years, median (IQR)^e^		33 (29–48)	36 (27–42)	0.428^a^	31 (28–37)	0.285^a^
BMI (kg/m^2^)^d^ (n, %)	BMI < 20 (9, 4%)	9 (100%)	0 (0%)	0.032^b^	0 (0%)	0.028^b^
	BMI 20–24 (77, 32%)	71 (92%)	3 (4%)		3 (4%)	
	BMI 25–29 (85, 33%)	63 (74%)	9 (11%)		13 (15%)	
	BMI ≥ 30 (51, 19%)^f^	38 (76%)	6 (12%)		6 (12%)	
Treatment(s) Received (n, %)	Orchidectomy + Chemotherapy (90, 35%)^f^	68 (76%)	10 (11%)	0.17^c^	11 (12%)	0.405^c^
	Orchidectomy alone (167, 65%)	140 (84%)	11 (6%)		16 (10%)	

^a^Significance level determined by Wilcoxon rank-sum test

^b^Significance level determined by Cochran-Armitage test for trend

^c^Significance level determined by Pearson’s chi-squared test

^d^BMI- Body mass index, ^e^ IQR- Interquartile range

^f^1 had unknown LH

Table 2 describes the clinical factors of interest associated with low total serum testosterone in Australian survivors testicular cancer.

Table 3 describes the clinical factors associated with primary low testosterone (vs normal testosterone) (*n*=229) and secondary low testosterone (vs normal testosterone) (*n*=235).

## Discussion

Our study reveals that low total serum testosterone was common among survivors of testicular cancer (17%), and there was a significant association between low testosterone and increasing BMI. While several factors may contribute to low testosterone levels, our findings suggest that higher BMI may influence testosterone levels more than age or chemotherapy use.

Hypogonadism is known to be associated with metabolic syndrome and increased cardiovascular risk, similar to the effect of androgen deprivation therapy on the development of insulin resistance and increased body fat mass observed in prostate cancer patients [23, 24]. An exploratory study involving 1135 Norwegian survivors of testicular cancer found that those receiving chemotherapy in addition to orchidectomy were approximately 3 times as likely to develop metabolic syndrome than those treated with surgery alone [3]. Serum total testosterone concentration was an independent predictor of metabolic syndrome, with the group receiving high doses of cisplatin exhibiting a lower mean testosterone concentration than the surgery-only group [3]. These findings, coupled with other published data, support the notion that treatment-induced, primary hypogonadism may contribute to the increased risk of cardiovascular risk factors and cardiovascular disease seen in survivors of testicular cancer, in addition to the direct vascular effects of cytotoxic therapy [3, 7, 13, 25–27].

Current guidelines recommend routine screening for testosterone deficiency during follow-up [20] and initiating testosterone replacement therapy (TRT) only for individuals with symptomatic testosterone deficiency (i.e., low libido, decreased morning erections, loss of body hair, low bone mineral density) and/or clear evidence of testicular failure [21, 28]. Older studies have suggested that long-term TRT could increase the risk of cardiovascular disease, prostate cancer, polycythemia, blood clots, obstructive sleep apnoea, and infertility [21, 29–32]. However, a recent randomised controlled trial demonstrated that TRT was not associated with increased incidence of major cardiac events, although this trial focused on a much older (median age 63 years), non-cancer population [33]. In summary, the benefit of TRT for low testosterone levels due to conditions such as obesity or type 2 diabetes in cancer survivors is uncertain, and there is limited long-term safety data [21]. Guidelines recommend addressing reversible causes prior to initiating TRT in certain conditions that cause hypogonadism, such as obesity [28].

Furthermore, our study showed that 60% of survivors of testicular cancer were overweight or obese, as defined by measured by a BMI of 25 kg/m^2^ or more. This highlights the need to address broader health issues to ensure the long-term well-being of testicular cancer survivors.

Exercise is an increasing feature of cancer survivorship and may be key to mitigating some of these problems in survivors of testicular cancer [34]. Multiple clinical trials have emphasised the beneficial effects of exercise in improving functional, physical, and psychosocial outcomes for cancer patients, including survivors of testicular cancer [35–39]. Incorporating 30 min of aerobic activity three times a week has been shown to alleviate common adverse effects associated with cancer diagnosis and treatment, such as mood disturbances, fatigue, and reduced physical function [40, 41]. Additionally, there is evidence that exercise may improve the survival of adults diagnosed with breast, colorectal, or prostate cancer. While the evidence may not yet be definitive, the established benefit of increased physical activity warrants its recommendations for all cancer survivors [35, 36].

Prior publications have reported associations between testicular cancer treatment and the subsequent hypogonadism. However, our data suggests that pre-existing obesity is associated with both primary and secondary low total serum testosterone. While causal relationships cannot be proven by this retrospective study, the observations add to our understanding of secondary low total serum testosterone in this setting.

There are two recognised mechanisms for low total serum testosterone. One involves gonadal insufficiency post-orchidectomy, possibly contributed to by chemotherapy, resulting in primary low total serum testosterone. The other mechanism arises from the metabolic effects of chemotherapy and other factors, perhaps in conjunction with pre-existing obesity, leading to secondary low total serum testosterone due to the disruption of the hypothalamic-pituitary axis.

Previous data indicating age-related increases in hypogonadism come from the general population. Our data did not show evidence of an association of older age with low total serum testosterone in our cohort of survivors of testicular cancer, though this may be due to the relatively limited sample size and the fact that most of the patients in the cohort were young (< 40 years) and the oldest patient was 64 years old.

### Limitations of study

As a retrospective observational study, our analysis has inherent limitations. Firstly, it relies on a relatively small sample size, which was augmented by the inclusion of 53% of our cohort recruited from a prospective clinical trial, allowing us to leverage prospectively entered real-world data.

Our data lacked details about stage and treatment, including the type of chemotherapy, its intent (adjuvant or definitive), and the number of cycles administered. As such, we were unable to examine associations between treatment intensity, or extent of disease and low total serum testosterone.

There was also inconsistency regarding the number of measurements patients had of their hormone levels within the clinically relevant window. While the majority of the Chemocog cohort (135/151, 89%) had multiple measurements, the iTestis cohort had only one reading so a single measurement was used for this study. Although largely the selected measurement in the Chemocog cohort fell close to 6 months in line with standard surveillance and the iTestis cohort, reliability in diagnosing hypogonadism and establishing a temporal trend between testicular cancer treatment and hypogonadism would benefit from analysing multiple assessments rather than relying on a single testosterone and LH level measurement.

Moreover, not all hormone measurements were conducted in the morning when testosterone levels are at their highest, potentially overestimating the incidence of low testosterone due to physiological diurnal variation. Our study also did not collect information regarding the incidence of TRT within our cohort, which would have influenced measured testosterone levels.

Furthermore, the analysis did not account for other predicting factors associated with the risk of secondary hypogonadism, such as steroid use or type 2 diabetes. While these comorbidities are unlikely in this cohort of young men, including these baseline variables, which are independent risk factors for hypogonadism, would have strengthened the analysis.

Additionally, we did not have longitudinal follow up of body weight and/or BMI over time. Most weights and heights were taken from anaesthetic assessments prior to orchidectomy or chemotherapy charts, however some were obtained following treatment completion. As such, the onset of low total serum testosterone in relation to the onset time of obesity was unable to be determined.

Another potential limitation is our use of BMI as the sole predictor of obesity. While there is growing evidence supporting waist circumference as a superior predictor of cardiovascular mortality and morbidity, particularly in individuals with a high muscle content [36], waist circumference is not typically recorded in clinical trials or routine practice.

## Conclusions

Low total serum testosterone was prevalent in our cohort of Australian survivors of testicular cancer. Low total serum testosterone is associated with reduced sexual function, low mood, cardiovascular disease, and potential deleterious impacts on quality-of-life and deserves our attention. We demonstrate a significant association between obesity and both primary and secondary low total serum testosterone, suggesting that obesity may play a significant role in driving the pathogenesis of this condition. Moreover, we also highlight the prevalence of increased BMI in this young male population. In addition to routine active surveillance, clinicians treating testicular cancer should consistently address lifestyle factors, particularly emphasising weight management and exercise programs in survivorship care. This approach may address low total serum testosterone, but regardless, will optimise the overall well-being of this expanding group of individuals.

## Data Availability

No datasets were generated or analysed during the current study.

## Abbreviations

ANZUP: Australian and New Zealand Urogenital and Prostate Cancer Trials Group
BMI: Body mass index
FSH: Follicular stimulating hormone
IQR: Interquartile range
LH: Luteinising hormone
T: Testosterone
TRT: Testosterone replacement therapy
